# Phase separation during blood spreading

**DOI:** 10.1038/s41598-021-90954-5

**Published:** 2021-06-03

**Authors:** Houssine Benabdelhalim, David Brutin

**Affiliations:** Aix Marseille Univ, CNRS, IUSTI, Marseille, France

**Keywords:** Applied physics, Biological physics, Fluid dynamics

## Abstract

Blood pools can spread on several types of substrates depending on the surrounding environment and conditions. Understanding the influence of these parameters on the spreading of blood pools can provide crime scene investigators with useful information. The focus of the present study is on phase separation, that is, when the serum spreads outside the main blood pool. For this purpose, blood pools with constant initial masses on wooden floors that were either varnished or not were created at ambient temperatures of $21~^{\circ }\hbox {C}$, $29~^{\circ }\hbox {C}$, and $37~^{\circ }\hbox {C}$ with a relative humidity varying from 20 to 90%. The range $21~^{\circ }\hbox {C}$ to $37~^{\circ }\hbox {C}$ covers almost all worldwide indoor cases. The same whole blood from the same donor was used for all experiments. As a result, an increase in relative humidity was found to result in an increase in the final pool area. In addition, at the three different experimental temperatures, the serum spread outside the main pool at relative humidity levels above 50%. This phase separation is more significant on varnished substrates, and does not lead to any changes in the drying morphology. This phenomenon is explained by the competition between coagulation and evaporation.

## Introduction

When a colloidal suspension is poured on a horizontal substrate, it will spread until reaching an equilibrium, which is characterized by wetting area and wetting contact angle^1,2^. In the case of porous substrates, more than the spreading, some liquid will permeate through the pores. At equilibrium, the final shape varies according to the initial volume and the involved forces^1,2^. The most encountered are spherical cap shape (drop, droplet), pancake shape (puddle, pool), and film. The last one represents the total wetting with a $0^{\circ }$ contact angle. In an unsaturated medium, simultaneously with spreading, volatiles liquid evaporate. This drying process leads to a sol-gel phase change^3^, and subsequently to cracks nucleation, which results from the competition between the adhesion to the substrate and evaporation^4^. As consequence, a characteristic dried pattern will form. It is known that the morphology of this pattern reveals information about liquid composition, involved phenomena in the drying process, and how the pattern was created. The spreading and drying are depending on the surrounding environmental conditions, substrate nature, and suspension concentration.

Human whole blood is assumed to be a colloidal suspension, where the plasma represents the liquid part, and cellular components are the colloids. In 2011, Brutin et al.^5^ showed that blood drops drying follows a similar mechanism as colloidal suspensions and depends on the blood composition, hence the individual health. Further, they give an exhaustive explanation about dried pattern formation. Bouzeid et al.^6^ studied the effect of relative humidity (drying rate) on the spreading dynamics and final pattern of the blood drop, and they showed that wetting areas increase with relative humidity. Also, the relative humidity influences the final pattern^7,8^, since the cracks density decrease with relative humidity. The substrate nature has an important role in the formation of the final pattern, as the evaporation rate on the drop surface and the flow inside the drop, since both are a function of the wetting contact angle^9^.

Understanding the physical and biological mechanisms underlying the formation of blood patterns is of great interest for forensic and biomedical applications. In the field of biomedicine, small blood drops are created on clean glass, and the dried patterns of the drop are used as a disease detector^5,10,11^. In forensic science, different types of bloodstains are often encountered, the most common being drip stain, cast-off pattern, and pool^12^. Through the use of Bloodstain Pattern Analysis (BPA), based on the fundamental principles of physics, chemistry, and biology, investigators are able to determine how, where, and when a crime occurs. In the literature, we can find two reviews on the formation of bloodstains from a physical and biological point of view^13,14^. In which, the relationship between fluid dynamics and BPA is considered, highlighting the significant contribution of fluid dynamics in the understanding of bloodstain formation^14^. Additionally, the wetting and spreading of human blood are also discussed^13^.

This study focuses on the characteristics of the spreading and drying of blood pools under different environmental conditions, particularly phase separation. This phenomenon is commonly encountered at crime scenes, such as in the well-known case of David Camm^15^. Blood pool represents an accumulation of blood on a substrate^12^. Laan et al.^16^ divided the drying morphology of blood pools into five distinct stages: spreading, coagulation, gelation, rim desiccation, and final desiccation. During drying, the mass loss is similar and reproducible for blood pools created under various conditions^16,17^. Using the third and fourth stages of blood pool drying, that is, desiccation, Smith et al. developed a patented model for determining the time at which a blood pool was created^17^. In another study, Laan et al.^18^ showed that blood pools containing an anti-coagulant dry in a different manner comparing to pools of pure blood. In addition, it is also worth mentioning the work of Ramsthaler et al. on the drying of drops and pools of blood^19^, as well as the work of Laber and Epstein on the effect of the nature of the substrate on blood coagulation time^20^. The drying of human whole blood, collected from healthy donors, is not dependent on donor age and pool volume. Laan et al.^16^ in their study about the morphology of pool drying, showed that blood pools dry in the same manner. Further, Smith et al.^17^ demonstrate that the volume and geometry of the pool do not affect the drying morphology.

Phase separation occurs when the serum spreads outside the main blood pool. In previous studies, this phenomenon has been found to be related to the inclination of the substrates in the surrounding environment^19,20^, and occurs only at blood volumes greater than 10ml^19^.

Little is currently known about phase separation during the spreading of the blood pool. To investigate this phenomenon and understand the mechanism that triggers it, the spreading and drying of blood pools were studied using the same volume of blood (4.6 ml ± 0.5%), i.e. less than 10 ml. Under different temperatures and relative humidity, we assumed that phase separation could be triggered by external parameters. Blood pools were created on two types of substrates: smooth (varnished) and rough (not varnished). The blood used in this study was obtained from the same healthy volunteer, with a hematocrit of 42.8% ± 2.6%, and the pools were created within 2 min post-collection to simulate a real crime scene.

In this study, the results on phase separation are presented as a function of external conditions (temperature, relative humidity, and substrate nature). Serum was found to spread out from the main pool under specific conditions. For example, a high relative humidity was found to increase this separation, as well as the use of smooth substrates, in contrast to rough substrates and a low relative humidity. In addition, the surrounding conditions and phase separation are investigated in terms of their effects on the spread of the total blood pool, the final blood pattern, and the visual drying time.

## Materials and methods

The whole human blood used in our study was collected by a certified nurse from a single donor to avoid any effects of blood composition. The donor was a 29-year-old healthy male; he did not take any medication or exert any physical effort prior to blood collection, as this can trigger an immune system response, resulting in changes in the blood composition. The blood used to create the pools was collected in two neutral 9 ml-tubes without anticoagulant or activator (Greiner Bio-One 9 ml Vacuette tubes). In addition, 3 ml-tubes covered with an anticoagulant (Greiner Bio-One 3 ml Vacuette tube) were used to collect blood for use in hematological blood analysis. The hematocrit level was determined using an analyzer (Mindray, BC 3600). The average hematocrit level across the experiments was $42.8\%~\pm ~2.6\%$.

**Figure 1:**
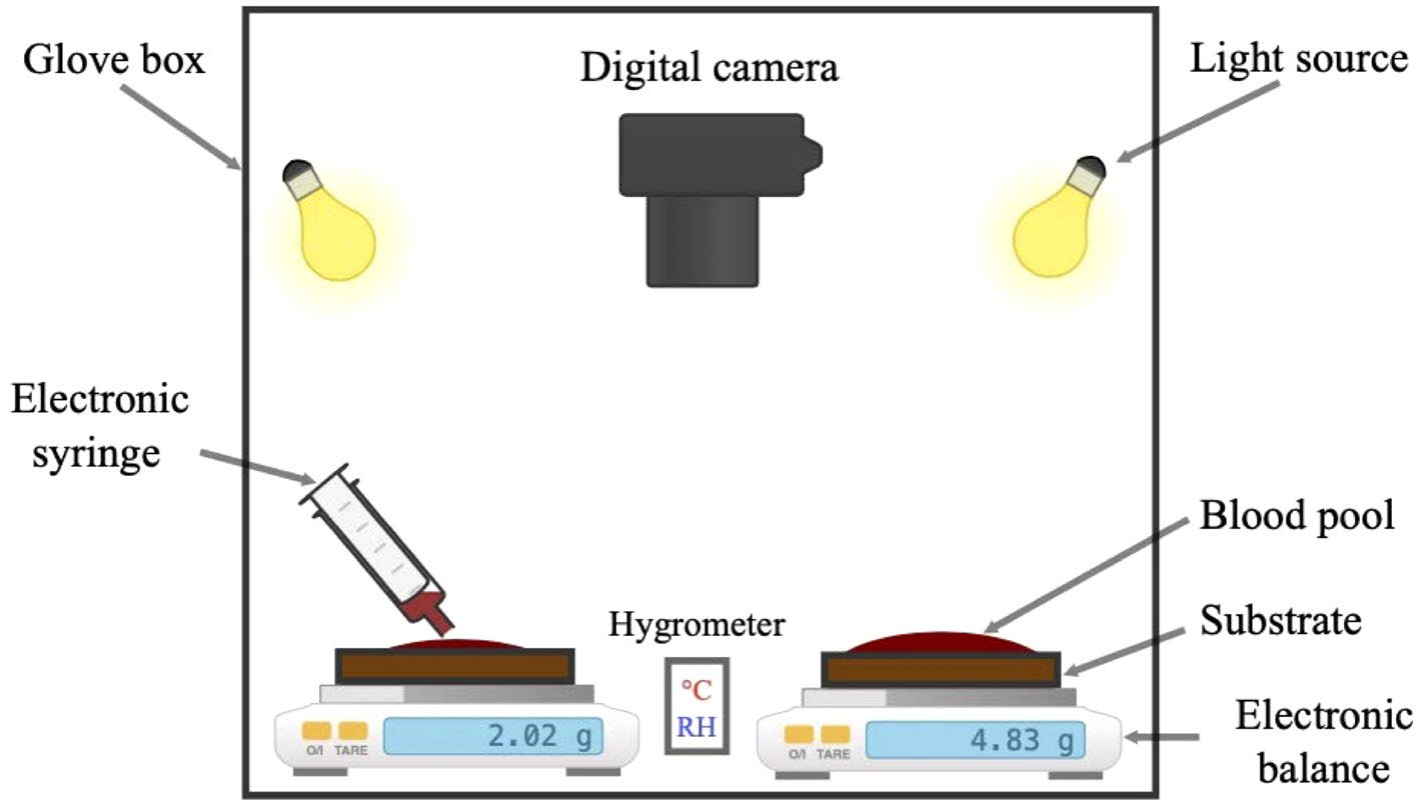
Experimental setup inside the glove box, with a relative humidity control system.

To simulate a real crime scene, blood pools were created on the experimental substrates less than 2 min after blood collection to avoid coagulation. The initial mass of the pools was constant ($m_i = 4.82~$g$~\pm ~0.5\%$). Two different types of substrates were used: wooden floors without varnish and wooden floors with varnish (ref:69924414, Leroy Merlin, France). Varnished wooden floors were obtained by applying three layers of varnish (ref:816054, Quincaillerie Aixoise, France). The use of these two substrates allowed us to investigate the characteristics of phase separation on rough and smooth substrates. Blood pools were created inside a glove box (Jacomex T-Box, $V = 350$ L) Fig. 1 at a constant environment. The relative humidity (RH) was controlled through a regulator, varying from $20\%$ to $90\%$ with a $10\%$ step and an uncertainty of $\pm ~2\%$. The temperature of the room was regulated using an air conditioning system to maintain a constant temperature inside the glove box. The experiments were performed at temperatures of $21~^{\circ }\hbox {C}$, $29~^{\circ }\hbox {C}$, and $37~^{\circ }\hbox {C}$, with an uncertainty of $\pm ~1^{\circ }\hbox {C}$. This values are chosen because quit all temperature indoor are between $21~^{\circ }\hbox {C}$ and $37~^{\circ }\hbox {C}$.

**Table 1:** Mass and hematocrit level of the blood samples used in this study for each surrounding conditions.

Temperature ($^{\circ }\hbox {C}$) $\pm ~1^{\circ }\hbox {C}$	Relative humidity (%) $\pm ~2\%$	Hematocrit (%)	Substrate	Mass (g)
22.6	19.9	43.8	Varnished	4.84
			Unvarnished	4.81
21.0	30.3	44.1	Varnished	4.84
			Unvarnished	4.83
21.7	40.9	43.4	Varnished	4.82
			Unvarnished	4.79
21.4	50.0	42.4	Varnished	4.83
			Unvarnished	4.82
21.3	60.1	41.5	Varnished	4.82
			Unvarnished	4.89
21.5	71.5	41.1	Varnished	4.83
			Unvarnished	4.79
23.4	81.3	43.8	Varnished	4.83
			Unvarnished	4.82
22.5	88.0	44.4	Varnished	4.84
			Unvarnished	4.83
29.3	19.9	43.1	Varnished	4.82
			Unvarnished	4.81
29.0	30.4	42.5	Varnished	4.82
			Unvarnished	4.79
29.2	40.0	42.0	Varnished	4.83
			Unvarnished	4.82
29.7	50.7	43.5	Varnished	4.84
			Unvarnished	4.82
30.4	60.7	42.7	Varnished	4.85
			Unvarnished	4.82
30.6	72.0	41.2	Varnished	4.82
			Unvarnished	4.81
37.4	20.1	43.6	Varnished	4.82
			Unvarnished	4.81
37.8	30.6	42.9	Varnished	4.82
			Unvarnished	4.81
37.5	40.4	42.3	Varnished	4.82
			Unvarnished	4.84
37.6	50.1	42.0	Varnished	4.83
			Unvarnished	4.81
37.5	59.8	43.5	Varnished	4.89
			Unvarnished	4.90
37.5	75.4	42.3	Varnished	4.84
			Unvarnished	4.83
Average value		42.8% ± 2.6%	4.82 g ± 0.5%	

To compare the spreading dynamics, drying morphology, and phase separation, two blood pools were created at the same time on the two substrates. The temperature and the relative humidity were measured and controlled during the whole process using a thermo-hygrometer (Testo Saveris 2 H1) placed next to the two pools. The initial mass and the loss of mass during evaporation (drying) were measured using two scales (Mettler Toledo, MS6002TS). A Canon EOS 7D digital camera was installed above the pools and pictures were recorded every 2 min. By means of reference lengths placed next to the pools, the spreading kinetics and equilibrium surface area of the pools were determined using Fiji image processing software^21^. Table 1 summarise the different used blood samples in this study.

The evaporation of these two pools did not lead to saturation inside the glove box as the increase in the RH was $10\%$.

### Biological and physical properties of whole human blood

The whole human blood is composed of a cellular part (red blood cells, white blood cells, and platelets), and a liquid part (plasma), which consists of 92% water^22^.Red blood cells are the most abundant, they represent 97% of the volume of the bio-colloidal matter. Their volume percentage in the blood is given by the hematocrit level (hct). This latter varies between 38% and 50% for a healthy person. Outside the human body, blood naturally coagulates and forms clots. During this process, some components of the plasma will be consumed, and it becomes serum.

The whole human blood is a complex non-Newtonian fluid that can be considered as a colloidal suspension^23^. It is characterized by a shear-thinning behavior, where its dynamic viscosity decreases with the increase of the shear rate. At shear rates higher than 100 s$^{-1}$, blood behaves like a Newtonian fluid with a dynamic viscosity equal to 4 mPa s at a temperature of $37^{\circ }\hbox {C}$. The blood viscosity depends on the measurement method^23^, temperature^23,24^, and hematocrit level^23,25^. Harkness and Philips^23,24^ reported a decrease of 3% per $1~^{\circ }\hbox {C}$ of the dynamic viscosity with the increase of the temperature. However, the increase in the hematocrit level causes an increase in the dynamic viscosity, which is greater at low shear rates. Although, the serum has a Newtonian behavior with a constant value of dynamic viscosity at any value of the shear rate, which is equal to 1.23 mPa s under a temperature of $37~^{\circ }\hbox {C}$.

The surface tension of whole human blood and serum are described by a linear equation as a function of temperature in the range of $20~^{\circ }\hbox {C}$ to $40~^{\circ }\hbox {C}$^26^:

For whole blood

1 $$\sigma (t)=(-0.473\times T+70.105)\times 10^{-3}$$

For serum

2 $$\sigma (t)=(-0.368\times T+66.072)\times 10^{-3}$$

where $\sigma$, represent the surface tension on N/m, and *T*,represent the temperature on $^{\circ }\hbox {C}$.

### Ethics issues

Regarding ethical issues, we confirm that all protocols are performed at Aix-Marseille University in the frame of the French National CODECOH protocol that handle the use and conservation of human blood samples. We also confirm that all experimental protocols were approved by the ethics committees of Aix-Marseille university, and we have a specific ethical agreement. We confirm that the volunteers have signed an informed consent form for the blood withdrawal. We also confirm that the involvement of human participants in our research project follow the WMA Declaration of Helsinki as well as the relevant EU legislations, convention and declaration. Regarding the method, we confirm that all methods were carried out in accordance with the French National CODECOH guidelines and regulations. For example, the blood is sampled by a certified person only.

## Results

**Figure 2:**
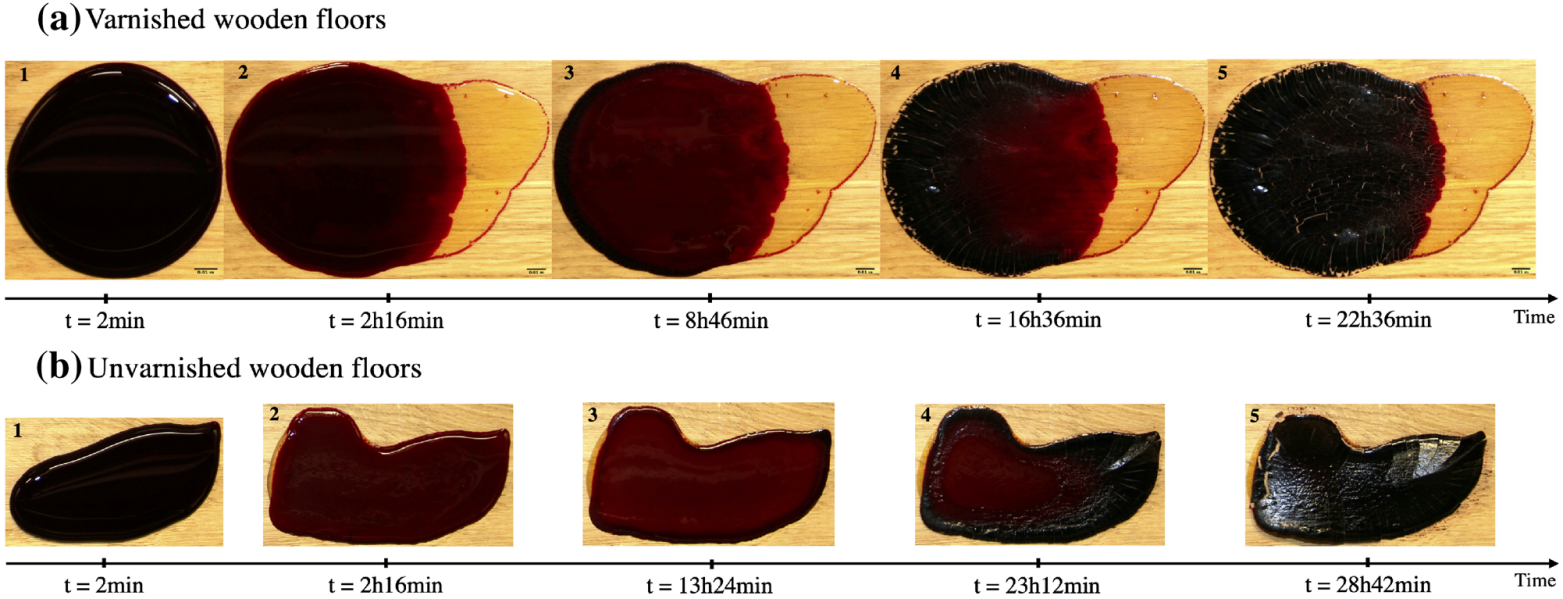
Time lapse of a drying pool of blood ($m_i = 4.82~g$, $hct = 41.5\%$) on (**a**) varnished and (**b**) unvarnished wooden floor at $21~^{\circ }\hbox {C}$ with a relative humidity of $60\%$. Serum separation and the five drying stages are shown.

### Effect of substrate nature on phase separation

The drying morphology of the blood pools has been previously described in detail by Laan et al., who divided this morphology into five distinct stages depending on the dominant phenomena^16^:

1. When pouring a given volume of blood on non-porous substrates, the blood first spreads and evaporates naturally before coagulating after several minutes. These phenomena characterize the first stage, during which the blood pool is dark red in color.
2. In the second stage, the color of the blood changes to a light red, and the front of the pool gels.
3. In the third stage, another front is formed characterized by a black color and the presence of small cracks; these two fronts spread towards the center of the pool as the pool dries.
4. In the fourth stage, the pool is completely gelled, evaporation holds through the pores, and the drying front continues to propagate towards the center.
5. In the fifth stage, the pool is completely dry, characterized by a black color and the presence of cracks. Under certain conditions, flakes may form, eventually becoming detached and separating from the pool.

The duration of each of these stages and the total drying time depend on the environmental conditions and on the nature of the substrates on which the pool is created. In their study, Laan et al. described the drying morphology of blood pools created on a linoleum substrate under a relative humidity of $32\%$. In the present study, several parameters were included and varied, such as relative humidity, temperature, and substrate nature.

**Figure 3:**
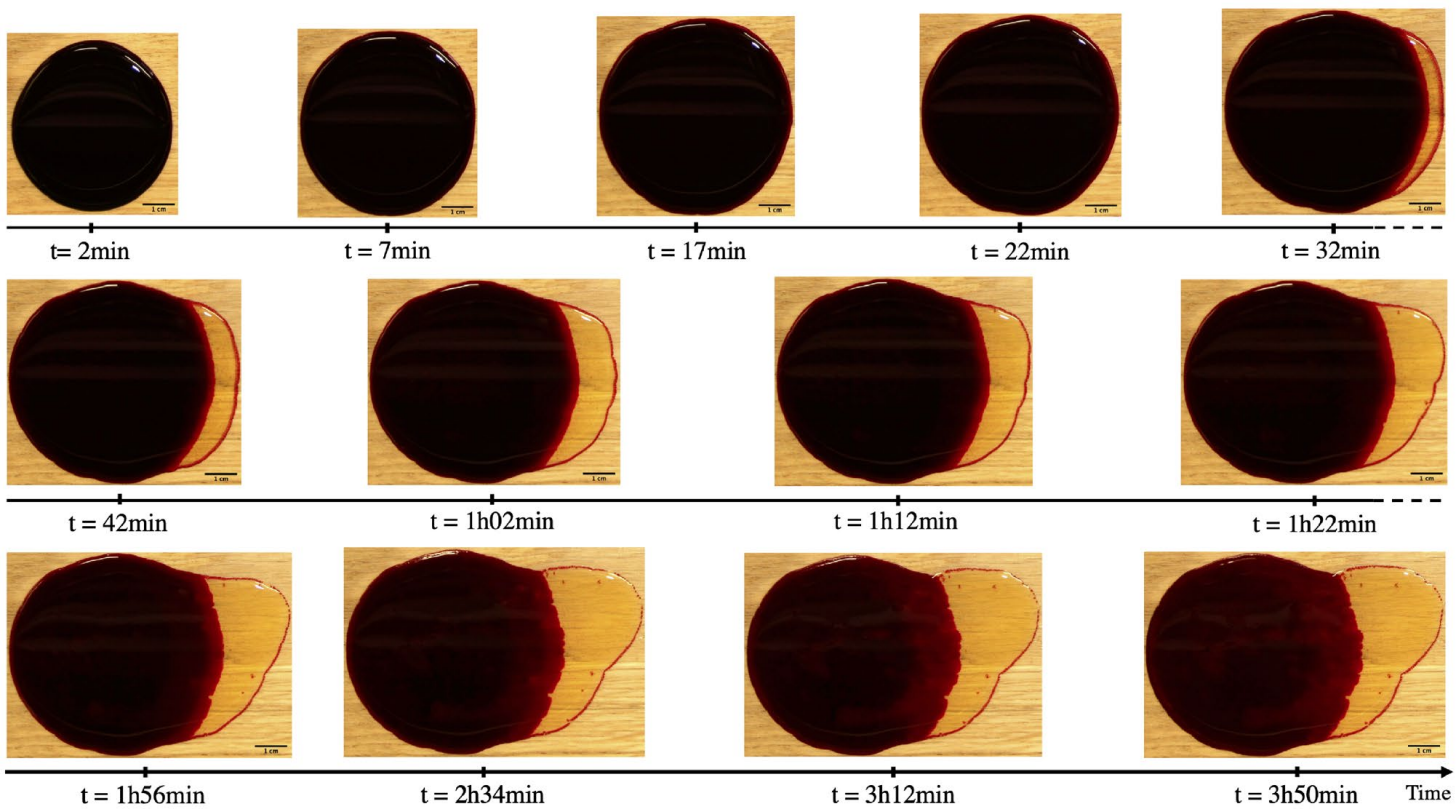
Time lapse of a drying pool of blood ($m_i = 4.83~g$, $hct = 41.5\%$) on varnished wooden floors at $21~^{\circ }\hbox {C}$ with a relative humidity of $60\%$, showing spreading and serum separation. For a movie of the drying blood pool, see the Supplementary Material.

Figure 2 shows the drying time of two blood pools created on wooden floors with and without varnish at a temperature of $21~^{\circ }\hbox {C}$ and a relative humidity of $60\%$. The final area reached at equilibrium was equal to 45.3 cm$^2$ in the case with varnished wooden floors and 20.1 cm$^2$ on unvarnished wooden floors. The equilibrium represents the complete halt of fluid flow during spreading. Phase separation can be observed in the images, represented by the yellow part spreading outside the main blood pool. This is the serum, which does not have the same components as the plasma, since part is consumed during coagulation. This separation is more significant on smooth substrates compared to rough ones, in which the serum only spread a small amount. Furthermore, despite phase separation, the drying morphology remained the same, namely in the existence of the five stages of drying described above. Serum separation was triggered 28 min after the creation of the blood pool on the varnished substrates, as shown in Fig. 3.

**Figure 4:**
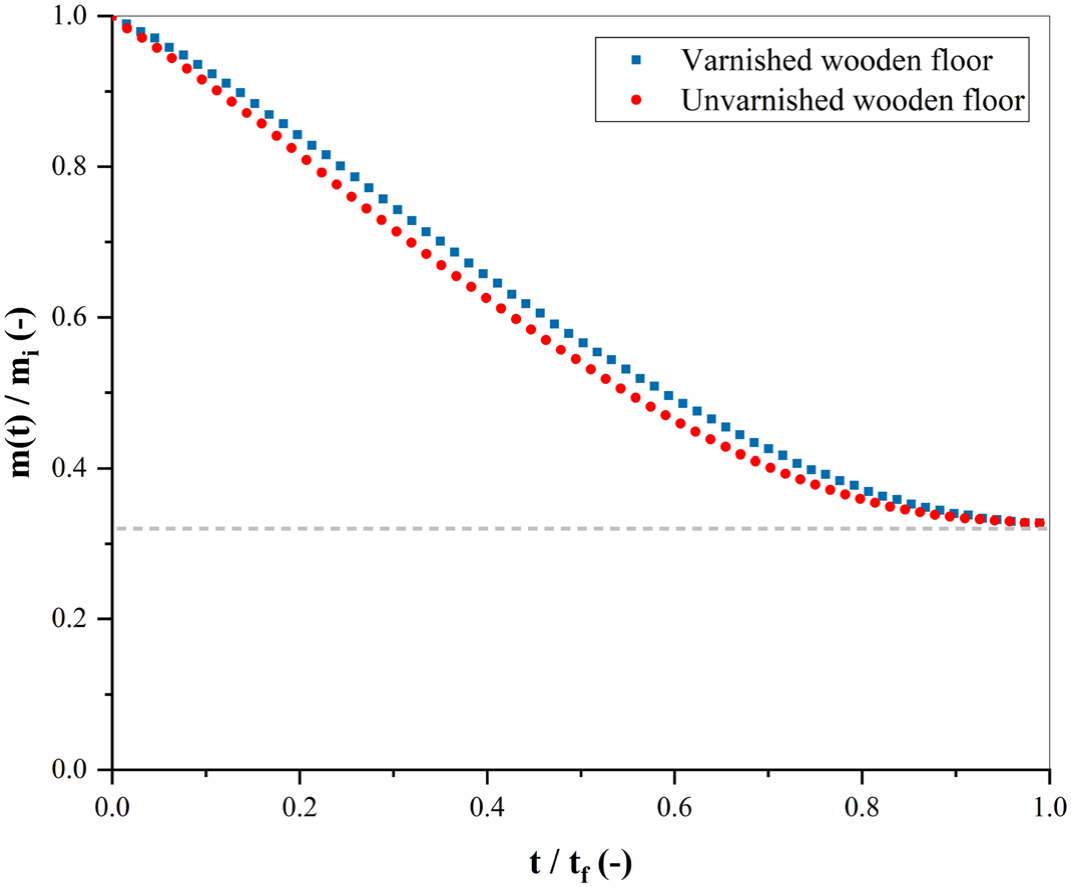
Drying kinetics of a blood pool ($m_i = 4.83~g$, $hct = 41.5\%$) on varnished (blue) and unvarnished (red) wooden floors at $21\,^{\circ }\hbox {C}$ with a relative humidity of 60%. The gray line represents the final ration $m_f/m_i =$ 32.3%. (OriginPro 2018).

The mass loss of these two pools is represented in Fig. 4, in which we plot the normalized mass as a function of normalized time $m (t) /m_i = f(t/t_f)$, where $m_i$ is the initial mass, $t_f$ is the final drying time, and $m_f$ is the final mass. These two latter are described as the point when there is no mass change, hence the end of the process. At the beginning of drying, the rate of mass loss (or evaporation rate) is not the same between the two substrates, this is due to the difference in the pool area. However, after approximately 20% of the total drying time, both pools exhibit the same mass loss rate until reaching 70%. In this time part, the pool is pinned to the substrate, and the drying takes place through the pores of gelled phase. At the end of drying, so after 70%, the rate decreases until the final mass, which corresponds approximately to 32.3% of the initial mass (gray line Fig. 4). Knowing that the non-volatile blood components represent 19% to 23% from the total mass according to the blood composition^22^, and Smith et al.^17^ show that the residual mass in dry conditions ($RH~<~30\%$) is equal to 23%. Indeed, at $RH~=~60\%$, an amount of liquid remains in the pool. The final mass of a dried pool depends strongly on the relative humidity. At $RH~=~0\%$ all the water evaporates, and at $RH~=~100\%$ there is no evaporation, hence no change in the mass. Between these two extreme values, the final amount of water in the blood pool increases with RH.

**Figure 5:**
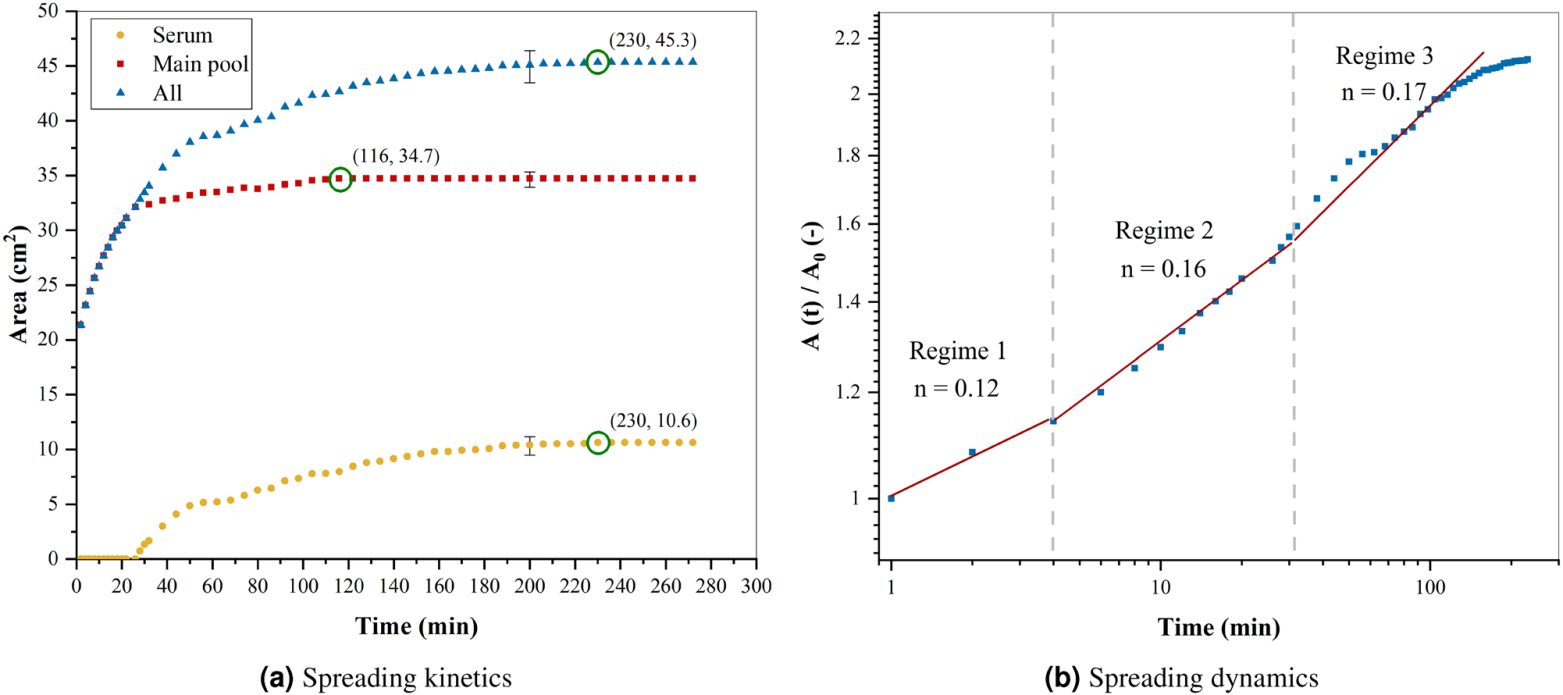
(**a**) Spreading kinetics of a blood pool ($m_i = 4.82~g$, $hct = 41.5\%$) on varnished wooden floors at $21~^{\circ }\hbox {C}$ with a relative humidity of $60\%$. (**b**) Log-log plot of the time evolution of the rescaled total pool area at $21~^{\circ }\hbox {C}$ with a relative humidity of $60\%$. The red line is a guide for the eyes, with three different values of slope.A typical error bar is presented on a single point of each curve. (OriginPro 2018).

Figure 3 shows a time lapse of the same pool shown in Fig. 2 on varnished wooden floors, with an emphasis on the phase separation. Initially, the blood pool spread and evaporated at the same time. Then, after 28 min, during the first drying stage, the serum also started to spread outwards, at which time the main blood pool (red part) and blood serum spread together. Then, the fluid flow of the red part of the blood pool stopped and the serum continued to spread until equilibrium. The spreading kinetics of this pool are shown in Fig. 5a, where the yellow curve represents the serum, the red curve represents the main pool, and the blue curve represent the total pool (serum + main pool). When the serum started to separate, a change in the kinetics of the main pool was observed, namely a slowing down to a complete stop after 116 min. As a result, between these two critical times, the serum and the main pool did not have the same kinetics. Before reaching equilibrium, the serum spread until 230 min. After the separation, the main pool continue to spread a little bit.

When blood comes into contact with a substrate, it spreads out to minimize the system energy^2^. Its spreading dynamics and those of liquids, in general, are described by the classical method as the competition between the different forces that react in the system: inertial, gravitational, capillary, and viscous forces. For the volatile fluids, evaporation at the liquid surface generates another internal force, which is a function of the evaporation rate. The existence of a temperature gradient at the surface generates a surface tension gradient, which will trigger an internal flow known as Marangoni flow. Different regimes characterize the spreading dynamics, and each regime represents a competition between two distinct forces. The two dominated forces are determined by using dimensionless numbers. By balancing these two forces, the law of evolution of the radius during spreading is given by a power law:

3 $$R(t) \sim t^{n}$$

The coefficient *n* depends on the driving force and the nature of the dissipation that govern the regime. The spreading dynamics of pools (large volume and bond number) of Newtonian liquids are composed of three regimes: a first gravitational-inertial regime at the moment of the liquid pouring, a second gravitational-viscous regime in which the pool takes the shape of a pancake, and a last capillary-viscous regime before the total stop of the flow. In the second regime, gravity dominates the flow except in the area closest to the contact line.

The spreading of blood pools does not follow a perfect circular shape. Therefore, to study the spreading dynamics, we chose to follow the area evolution over time, which seems more logical. Fig. 5b represents the log-log plot of the area *A*(*t*) normalized by the initial area $A_0$ as a function of time. In this figure, three regimes describe a power-law evolution as we have excepted. A power exponent $n = 0.12$ describes the evolution of the first regime, an $n = 0.16$ for the second regime, and the last regime with $n = 0.17$. Indeed, the first regime in Fig. 5b corresponds to the end of the gravitational-viscous regime, since we start recording after about ten seconds of the creation of the pool. The gravitational forces drive the spreading, although the viscous forces resist the flow. The transition from the first to the second regime takes place after 3 min of the pool creation. At the moment of phase separation, so after 28 min, we have the third regime. In these two spreading regimes, the driving force is produced by the evaporation of the water to the atmosphere, and the viscosity represents the resistive forces. These regimes are discussed thoroughly in the “Discussion” section.

**Figure 6:**
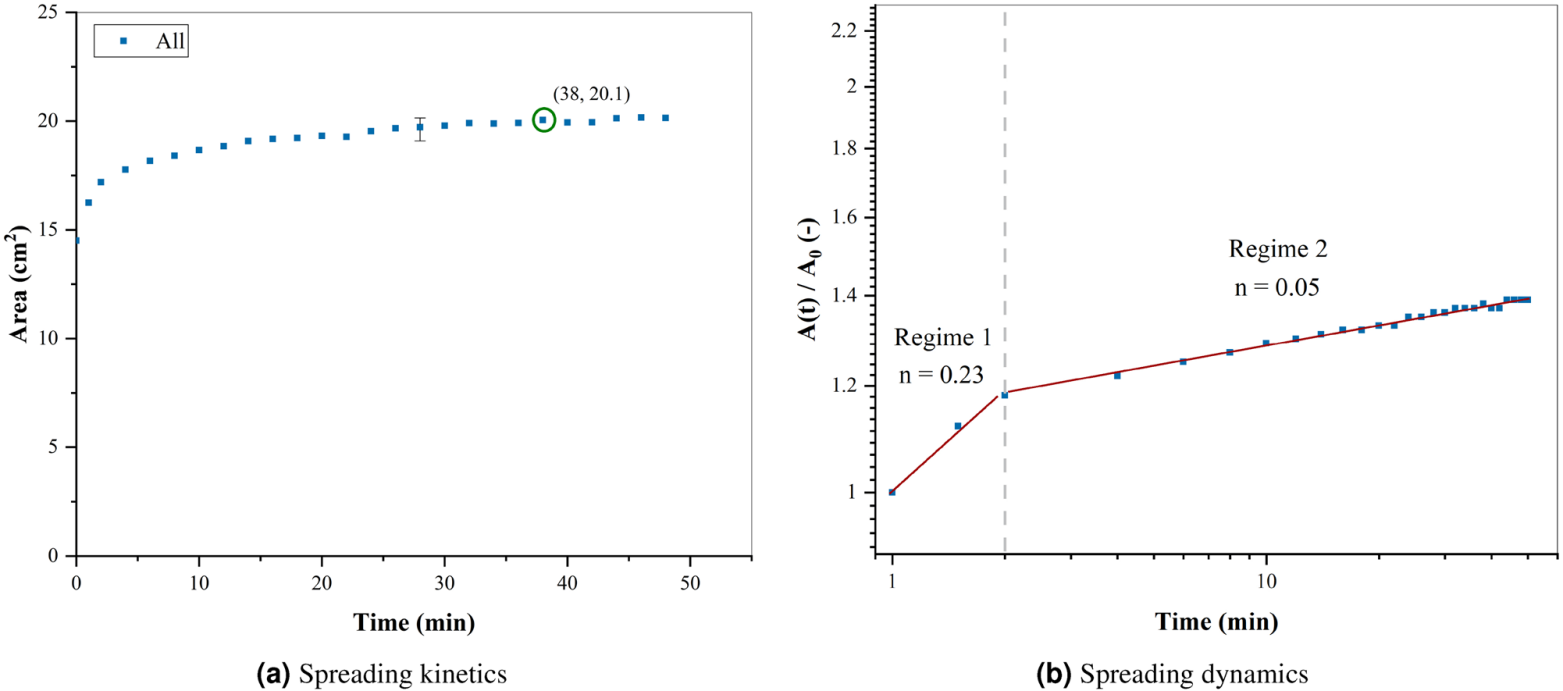
(**a**) Spreading kinetics of a blood pool ($m_i = 4.82~g$, $hct = 41.5\%$) on unvarnished wooden floors at $21~^{\circ }\hbox {C}$ with a relative humidity of $60\%$. (**b**) Log-log plot of the time evolution of the rescaled total pool area at $21~^{\circ }\hbox {C}$ with a relative humidity of $60\%$. The red line is a guide for the eyes, with two different values of slope. A typical error bar is presented on a single point of the curve. (OriginPro 2018).

Figure 6a shows the evolution of the area during the spreading A(t) and Fig. 6b the log-log plot of the area A(t) normalized by the initial area A0 as a function of time, of the blood pool on the unvarnished wooden floor, under the same conditions ($m_i = 4.82~g$, $hct = 41.5\%$, $T~=~21~^{\circ }\hbox {C}$, and $RH = 60\%$). Where the blue curve represents the total pool, since in this case the serum separates a small amount, and this does not affect the spreading dynamics. In Fig. 6a, the initial point is equivalent to the time on which the first picture was taken. The pool spread until reaching equilibrium after 38 min and a wetting area of 20.1 cm$^2$. As a result, the pool reaches equilibrium faster than on a varnished wooden floor, but the final area is lower. However, the spreading dynamics is characterized only by two regimes, a gravitational-viscous regime and viscous-evaporation rate equilibrium, with $n = 0.23$ and $n = 0.05$ respectively. The transition between these two regimes occurs after 2 min from the creation of the pool. These differences in the spreading dynamics between substrates are due to the roughness and the absence of phase separation.

These findings suggest that phase separation and equilibrium area depend on the nature of the substrate on which the blood pool is created. The ratio of the separated serum area was $23.1\%$ on the varnished wooden floors (smooth substrate) and $0.4\%$ on the unvarnished wooden floors (rough substrate).

### Effect of relative humidity on phase separation

**Figure 7:**
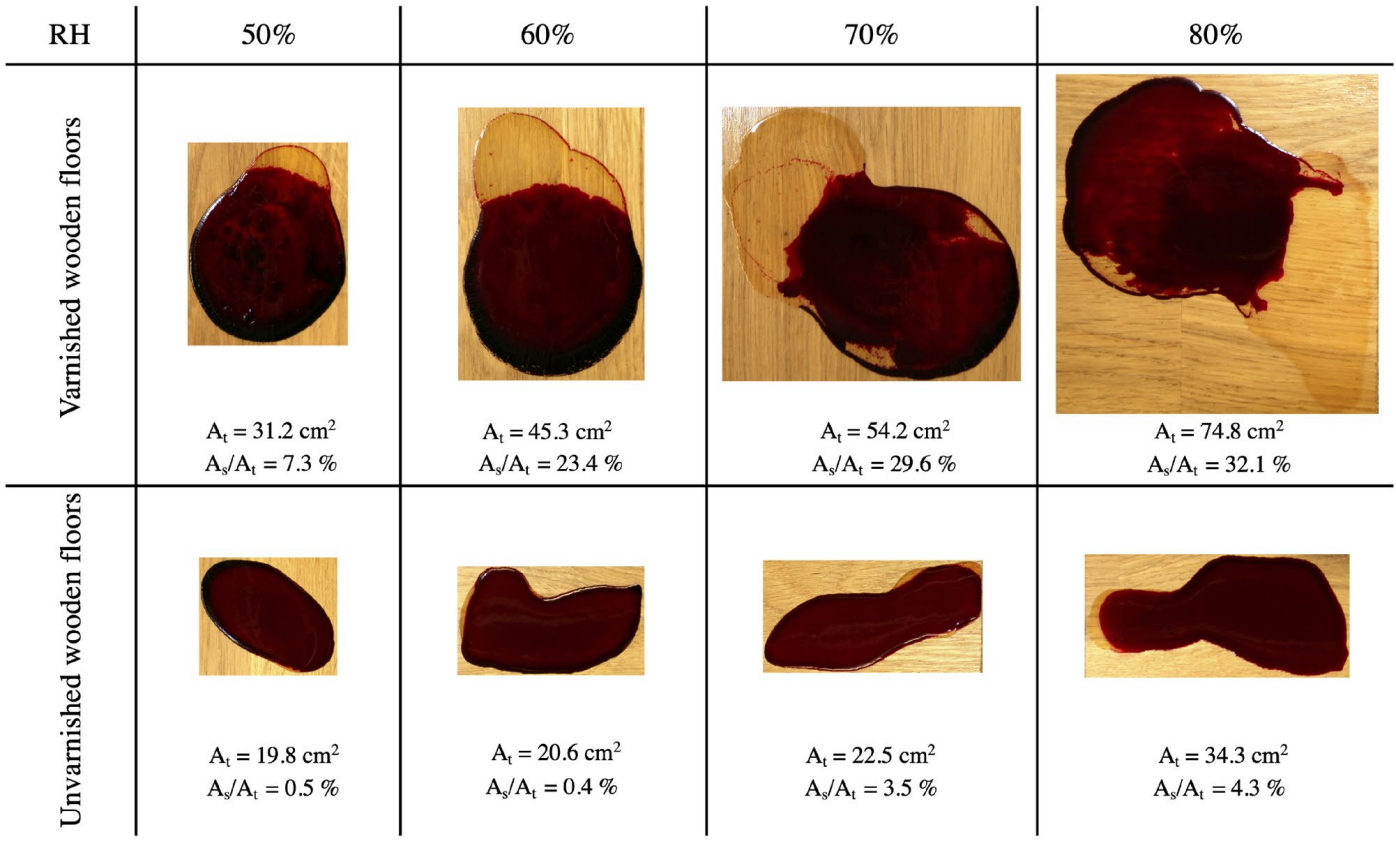
Effect of relative humidity and substrates on the spreading and phase separation of blood pools ($m_i = 4.82$ g$~\pm ~0.5\%$, $hct = 42.8\%~\pm ~2.6\%$). Pools created on varnished and unvarnished wooden floors at $21~^{\circ }\hbox {C}$ with relative humidities ranging from $50\%$ to $80\%$. $A_t$ denotes the total area of the pool and $A_s/A_t$ denotes the ratio between the serum area and the total pool area.

To confirm these observations and results, further experiments were carried out at a constant temperature of $21~^{\circ }\hbox {C}$ with different relative humidities. Figure 7 shows the blood pools created on varnished and unvarnished substrates under a relative humidity ranging from $50\%$ to $80\%$. As shown in the images, the increase in relative humidity led to an intensification of the phenomenon of phase separation, with a ratio of $7.3\%$ on the varnished wooden floors and $0.5\%$ on unvarnished wooden floors at $RH = 50\%$. At $RH = 80\%$, a ratio of $32.1\%$ on the varnished wooden floors and $4.3\%$ on unvarnished wooden floors was calculated. In addition, at different RHs, spreading and phase separation appeared to be more significant on varnished substrates than unvarnished substrates.

**Figure 8:**
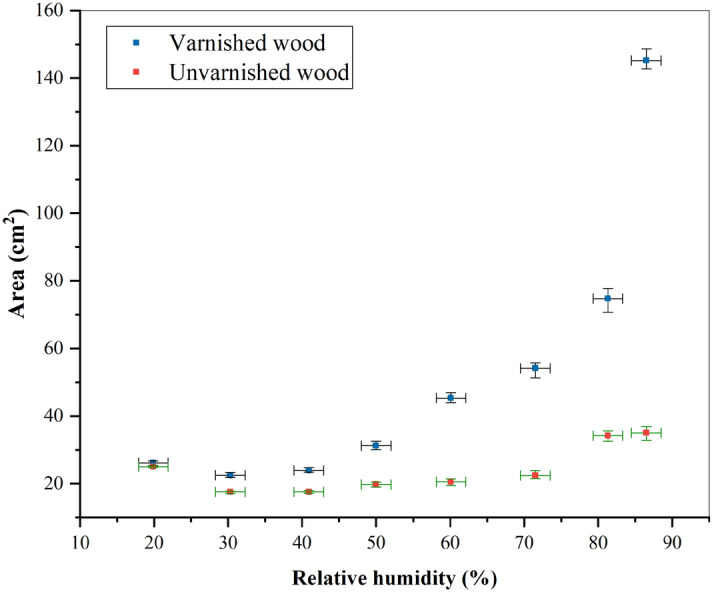
Total area of blood pools ($m_i = 4.82$ g$~\pm ~0.5\%$, $hct = 42.8\%~\pm ~2.6\%$) at equilibrium as a function of the substrate nature and relative humidity at a temperature of $21~^{\circ }\hbox {C}$. (OriginPro 2018).

Figure 8 shows the plots of the different final areas obtained at relative humidities ranging from $20\%$ to $90\%$ and a constant temperature of $21~^{\circ }\hbox {C}$. The thermo-hygrometer used had a relative humidity measurement error of $\pm ~2\%$; hence, the origin of the horizontal error bar. The variations in the contrast at the edge of the blood pool indicate a measurement error on the surface area, which is represented by the vertical error bar. Only small variations in the final area between the two substrates were observed at a low relative humidity, compared to a high relative humidity, in addition to the time needed to reach equilibrium and the change in spreading velocity. As a result, the blood pools spread significantly more with an increasing relative humidity.

**Figure 9:**
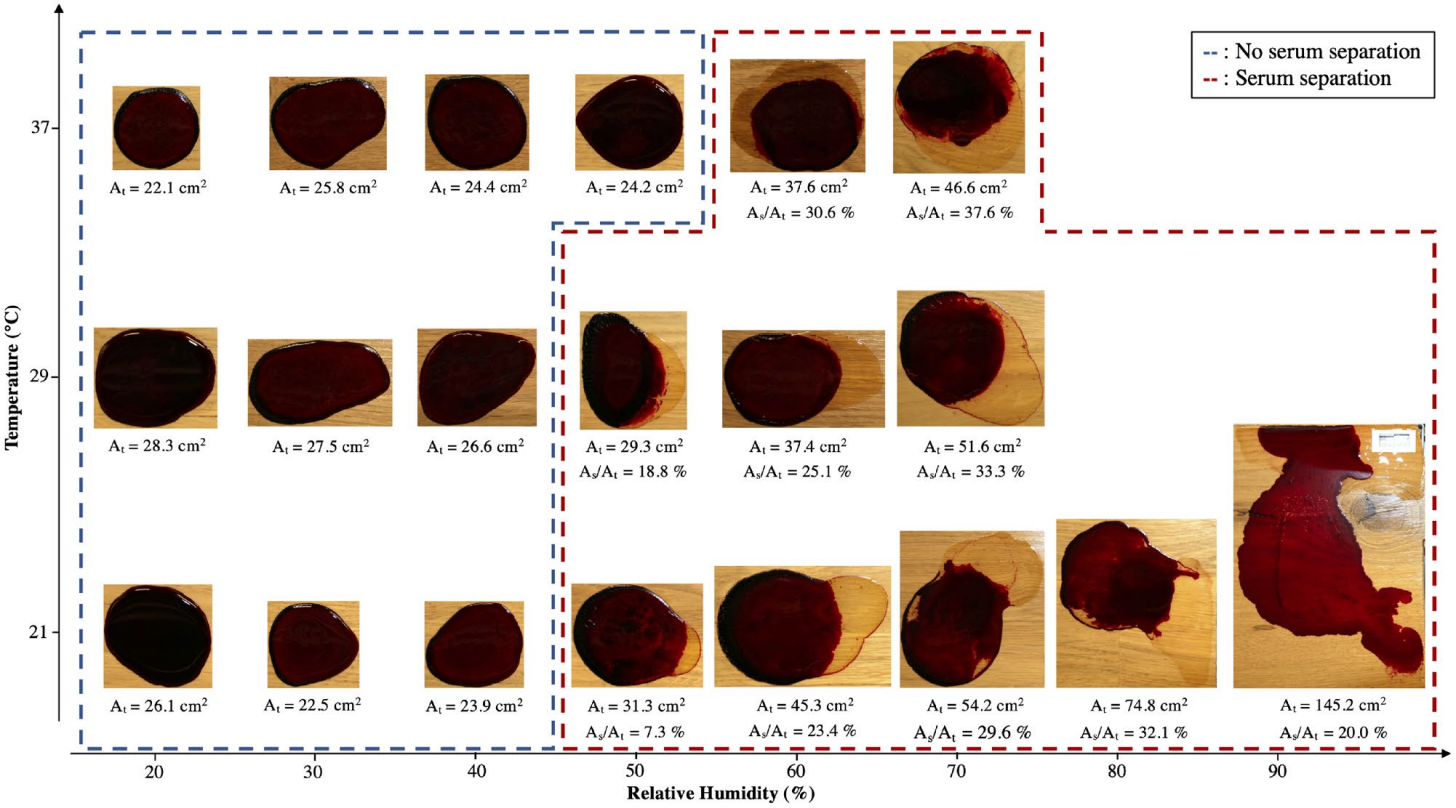
Phase separation during the spreading of blood pools ($m_i = 4.82~g~\pm ~0.5\%$, $hct = 42.8\%~\pm ~2.6\%$) as a function of relative humidity and temperature on varnished wooden floors. The blue box represents cases without phase separation, and the red box represents cases with phase separation. $A_t$ is the total area of the pool and $A_s/A_t$ is the ratio between the serum area and the total pool area.

To study the effect of the environmental conditions on phase separation further, the spread of experimental blood pools under a wide range of relative humidities and temperatures was investigating. In addition, the formation of blood pools was studied on different surfaces, including varnished wooden floors (smooth substrate), on which the separation was found to be more significant, providing important insights into this phenomenon. The results of these experiments are shown in Fig. 9. An increase in the relative humidity resulted in an larger final area at constant temperatures, as shown in Fig. 8 (blue points). In addition, the serum was found to separate from the main pool only at relative humidities over $50\%$, indicating the existence of a threshold phenomenon for healthy human blood with a hematocrit of $42\%$. In Fig. 9, the blue part represents blood pools without phase separation under a low RH, while the red part represents blood pools with phase separation under a high RH.

### Final pattern and adhesion

**Figure 10:**
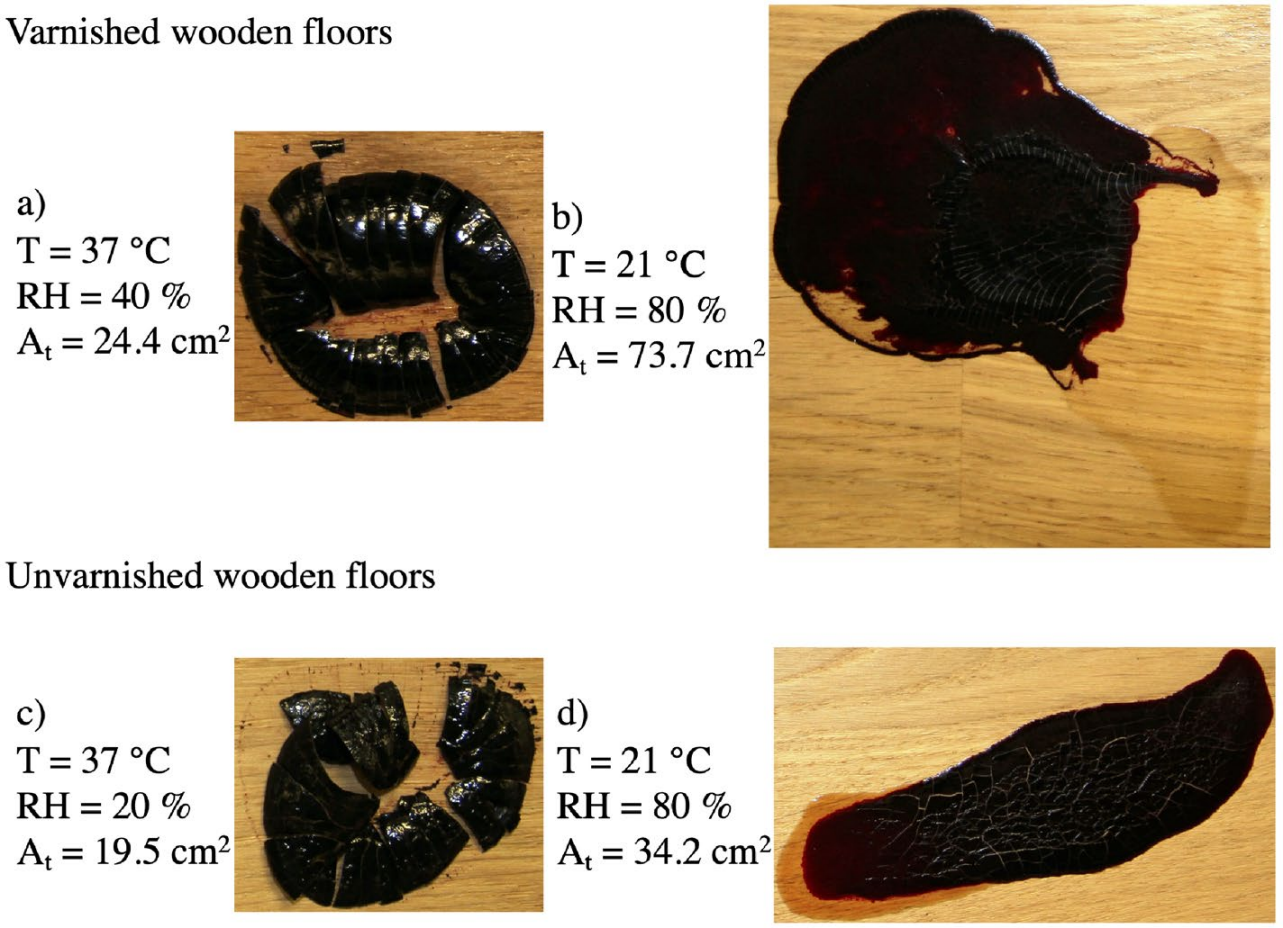
Final pattern of dried blood pools ($m_i = 4.82~g~\pm ~0.5\%$, $hct = 42.8\%~\pm ~2.6\%$) on varnished and unvarnished wooden floors as a function of relative humidity and temperature. $A_t$ is the total pool area.

At pattern emerging at the end of the drying stage can have different visual aspects, including the presence of cracks, delamination, and adhesion (Fig. 10). All of these aspects depend on the shape and size of the blood pools, the composition of the blood, the environmental conditions, and the nature of the substrates. Cracks were present in all 40 blood pools studied. The nature of the substrates and the relative humidity were found to be decisive factors in terms of adhesion and delamination. For example, on the unvarnished substrates, blood pools only adhered to the surface at relative humidities above $80\%$; otherwise, they delaminated by forming flakes at a lower RH. In the case of varnished substrates, this limit fel to $60\%$. Figure 10 summarizes the four possible cases obtained. For the same blood volume, adhesion was found to depend on the relative humidity. Fig. 10a,c represent the delamination of the completely dried pools under a low relative humidity on both substrates, while Fig. 10b,d represent the adhesion of the pools under a relative humidity of $80\%$. Upon phase separation, the serum was found to always adhere to the surface.

**Figure 11:**
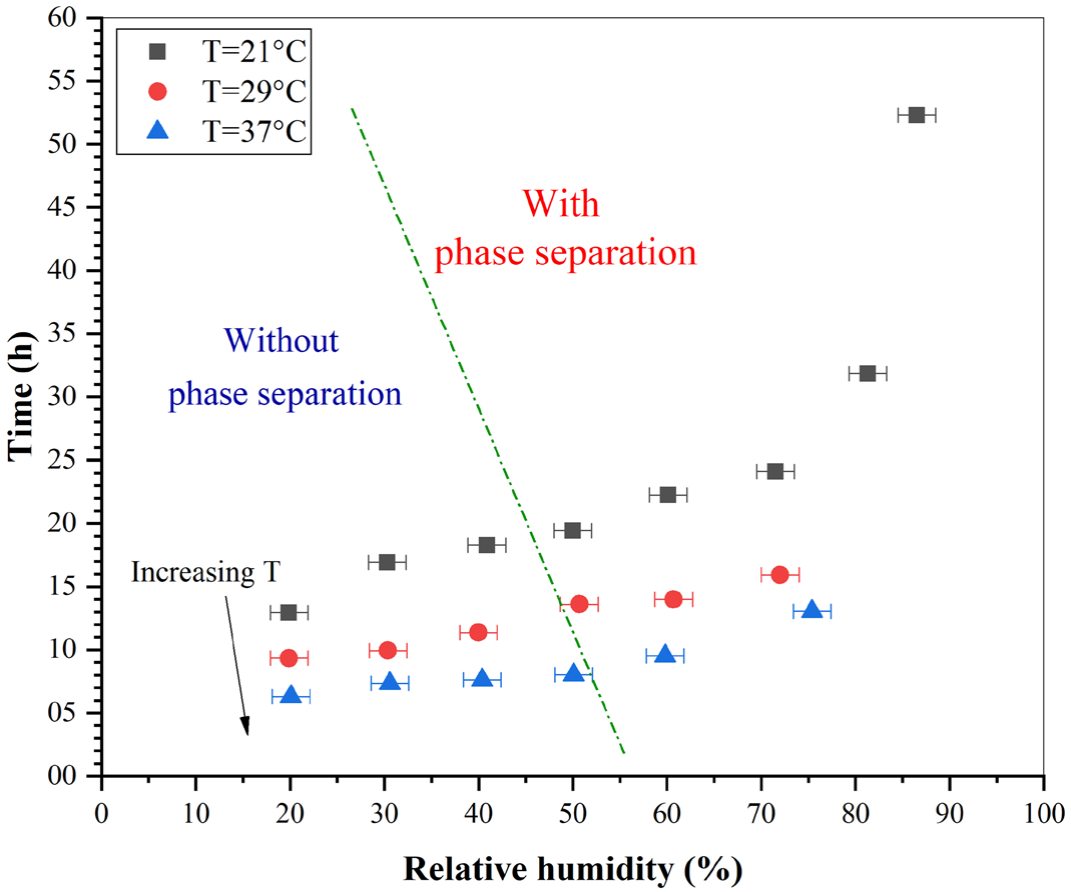
Visual drying time of blood pools ($m_i = 4.82~g~\pm ~0.5\%$, $hct = 42.8\%~\pm ~2.6\%$) on varnished wooden floors as a function of temperature and relative humidity. The green dotted line separates the cases with and without phase separation. (OriginPro 2018).

Temperature did not have a significant effect on adhesion. However, it did play an important role in the drying time. Figure 11 shows the visual drying time as a function of temperature and RH for pools created on varnished wooden floors, where time represents the time at which the pools become completely black. Drying time decreased with an increasing temperature, resulting in faster drying. By contrast, an increase in RH slowed down the drying process. The same pool was found to dry in 6h18min at $T = 37^{\circ }\hbox {C}$ and $RH = 20\%$ compared to 52h18min at $T = 21^{\circ }\hbox {C}$ and $RH = 90\%$. As shown in Fig. 11, despite the presence of phase separation, the curves maintained the same trend for the three temperatures.

## Discussion

The formation of blood stains at a crime scene can take place under many different environmental conditions, which can be either controllable or not, depending on the presence and activation of Heating, ventilation, and air conditioning system (HVAC system). In addition, blood pools can be created on different types of substrates. The spreading and wetting of fluids are defined by two important parameters: the final surface area and the contact angle at equilibrium, which are mainly a function of roughness, temperature, and relative humidity. In the case of ideal, smooth, and homogeneous substrates, the equilibrium contact angle is given by Young’s law. However, real surfaces are heterogeneous and rough, which affects their wetting characteristics, particularly the apparent contact angle. The latter is given by Wenzel’s model or that of Cassie-Baxter. In Fig. 7, under the same relative humidity, the equilibrium areas were found to change as a function of the substrates, namely a smooth substrate (varnished wooden floor) and a rough, little porous substrate (unvarnished wooden floor). With the same volume (mass) for all the blood pools, a difference in surface area necessarily led to the variations in the thickness of the blood pool and contact angle. Thickness h is given by the following approximation:

4 $$h=\frac{V}{A}$$

where *A* is the equilibrium pool area, and *V* is the pool volume. The contact angle is given by^17^:

5 $$\theta =arccos(\frac{g m^2}{2 \gamma A^2}+1)$$

with $\gamma$, surface tension, and *m*, the mass of the pool. The influence of roughness on the spreading and contact angle at equilibrium has been considered by several researchers^27,28^, who have shown that an increase in roughness leads to an increase in the maximum contact angle and a decrease in the final area. The results obtained and shown in Fig. 8 correspond perfectly to the theory: the varnished substrate represents the case with low roughness (smooth), on which the blood spread significantly more compared to the unvarnished substrate (rough and little porous).

To give an exhaustive explanation of phase separation, it is necessary to understand the spreading dynamics. The first regime in Fig. 5b corresponds to a gravitational-viscous regime with a power n equal to 0.12 $\sim$ 1/8. This evolution is the same as that obtained by Cazzabat et al.^29^ in their study on oil spreading. In our experiments, all blood pools have a pancake shape, which results from the fact that the gravitational forces are more important than the capillary forces, unlike for the drops, which have a spherical cap shape^2^. Also, in this regime, we have a Bond number greater than 1 and a characteristic length (the equivalent radius) higher than the capillary length.

When a blood pool is deposited on a given substrate in an unsaturated condition, the volatile component that is water begin to evaporate naturally. The existence of a difference in vapor concentration between the pool and the environment causes evaporation, resulting in a reduction in the pool mass to the total drying. The pool evaporates with a given evaporation rate, which is a function of humidity and temperature. The decrease in the pool mass is proportional to the evaporation rates. At a constant RH, the higher the temperature is, the higher the evaporation rate is, resulting in rapid drying. At a given temperature, the increase in RH leads to a decrease in the evaporation rate, resulting in a slower drying process with a longer spreading time.

In their study on the spreading of blood drops as a function of relative humidity under a temperature of $24~^{\circ }\hbox {C}$, Bouzeid and Brutin^6^ have shown that there are two characteristic regimes of the spreading dynamics. The competition between the viscous and capillary forces controls the first regime, in which they observe an evolution of the surface area with an exponent n equal to 0.65. They defined the second regime as the viscous-evaporation rate equilibrium with a spreading exponent $n = 0.19$. Also, they showed that at a low evaporation rate (high RH), the spreading time is more important. As a result, the final drop area increases with increasing relative humidity. Our results presented in Fig. 8 are consistent with the theory. At low evaporation rates, blood pools spread further before reaching equilibrium, as shown in Fig. 8. The surface area of a blood pool increases with relative humidity.

In Fig. 5b, the competition between evaporation rate (driving flow) and viscosity (resistance) leads the surface area to evolve as a power law with an exponent equal to 0.16 and 0.17 for the second and third regimes, respectively. The transition between these two regimes corresponds to the time of phase separation, after 28 min of blood pouring. The evolution of the surface area in the third regime is faster than in the second one because the serum viscosity is much lower than that of the whole blood one.

Taking into account another phenomenon allow us to describe the mechanisms related to this separation. A blood pool on a non-porous substrate coagulates and separates into two phases: liquid phase (serum) and cellular phase^22^. Coagulation is defined as an aggregation of cells into a single block. The separation inside the pool is characterized by the sedimentation of the cellular components due to their density, which is higher than that of serum^18^.

Coagulation is not an instantaneous phenomenon. In contrast, coagulation occurs step by step. The total coagulation time of human blood depends on the substrate on which the blood is deposited^20^ and the temperature^30^. Increasing temperature accelerates the formation of blood clots. Table 2 shows the coagulation time of blood pools as a function of the substrate nature.

**Table 2:** Total coagulation time as a function of substrate nature, which is obtained from five individuals^20^.

Substrate	Coagulation time (min:sec)	Average coagulation time (min:sec)
Blood tube	5:15–8:00	6:34
Human skin	8:30–12:00	9:52
Glass	10:40–17:50	14:02
Wool-polyster cloth	13:20–28:20	22:32
Paper	22:17–34:31	28:46
Wood	27:21–37:10	32:29

Blood coagulation is equivalent to the sol-gel transition^17^, during which there is a formation of a porous medium. The porosity and the size of the pores increase dramatically with relative humidity^31^. The phase separation of the blood pool shown in Fig. 3 starts after 28 min, which nearly corresponds to the average coagulation time on wood.

All the cellular components stop spreading at the end of coagulation, contrary to the serum. Since the evaporation rate is low at high relative humidity, so the pool mass slowly decreases. At the end of the coagulation, we have a significant amount of serum. This latter will continue to spread to reach the equilibrium, resulting in a phase separation Fig. 9. However, at low relative humidities, the decrease in serum mass is quick. Thus, at the end of coagulation, a smaller amount of serum is remaining. Also, the pores formed are smaller, and they will trap serum and slow down its flow to the surface. In Fig. 9, at $37~^{\circ }\hbox {C}$, we do not have the same phase separation threshold. The increase in temperature leads to an acceleration of evaporation and coagulation. This separation threshold changes according to the temperature.

The final pattern of the blood pools is characterized by the presence of cracks (Fig. 9), whose formation during drying is due to tensile stress. This results from the competition between evaporation and adhesion of blood pools to the substrate^32^ and depends on the thickness of the pool^33^. During evaporation, this stress increases, and is only relaxed by the formation of cracks. In addition, adhesion and delamination are two additional important visual aspects that characterize the final pattern of blood pools. As discussed in the Results section and in Fig. 9, on a non-porous substrate, roughness and relative humidity determine whether there is pool adhesion to the substrate. Indeed, the ratio of volatile components remaining in the pool at the end of drying depends on the RH. At a higher relative humidity, there are more residual volatile components, which are the main elements that adhere to the substrate.

## Conclusions

In this study, we investigated the phase separation phenomenon in blood pools, that is, the spreading of the serum outside the main blood pool. To this end, blood pools of whole human blood were created under experimental settings. The same volume of blood was used to create blood pools on varnished and unvarnished wooden floors under different environmental conditions. To remove the effects of the blood composition, blood was collected from a single donor. The influence of relative humidity on the spreading and drying of the blood pools was investigated. As a result, an increase in the RH was found to result in a decreased evaporation rate, thus a longer drying time and the wider spreading of the blood pools. This also led to the spreading of serum outside the main body of the blood pools. Phase separation was found to be significant on varnished wooden floors (smooth substrate), occurring at RH higher than $50\%$. Despite phase separation, the five distinct stages that characterize the drying of blood pools were observed. At the end of drying, the pools were found to adhere to the substrate at RH levels above a threshold value depending on the nature of the substrate.

We evidence that phase separation occurs due to a competition between coagulation and evaporation of water in the serum phase. When the temperature is high and humidity is low, the water of the serum phase evaporates much faster than coagulation takes place. Thus an important water amount of the serum phase is evaporated when coagulation end. consequently serum phase separation can not occurs due to the presence of the clot. At the opposite with low temperature and high humidity, when coagulation start there is a significant of remaining serum phase that can spread throw the clot in formation. This mechanism justify that phase separation occurs upon a threshold.

## Supplementary Information

Supplementary Information 1.

Supplementary Information 2.

## References

[1] Bonn D, Eggers J, Indekeu J, Meunier J, Rolley E (2009). Wetting and spreading. Rev. Mod. Phys..

[2] Oron A, Davis SH, Bankoff SG (1997). Long-scale evolution of thin liquid films. Rev. Mod. Phys..

[3] Bodiguel H, Doumenc F, Guerrier B (2010). Stick- slip patterning at low capillary numbers for an evaporating colloidal suspension. Langmuir.

[4] Giorgiutti-Dauphiné F, Pauchard L (2018). Drying drops. Eur. Phys. J. E.

[5] Brutin D, Sobac B, Loquet B, Sampol J (2011). Pattern formation in drying drops of blood. J. Fluid Mech..

[6] Bouzeid W, Brutin D (2014). Effect of relative humidity on the spreading dynamics of sessile drops of blood. Colloids Surf. A.

[7] Bouzeid W, Brutin D (2013). Influence of relative humidity on spreading, pattern formation and adhesion of a drying drop of whole blood. Colloids Surf. A.

[8] Choi J, Kim W, Kim H-Y (2020). Crack density in bloodstains. Soft Matter.

[9] Chen R, Zhang L, Shen W (2018). Controlling the contact angle of biological sessile drops for study of their desiccated cracking patterns. J. Mater. Chem. B.

[10] Chen R, Zhang L, Zang D, Shen W (2016). Blood drop patterns: formation and applications. Adv. Colloid Interface Sci..

[11] Bahmani L, Neysari M, Maleki M (2017). The study of drying and pattern formation of whole human blood drops and the effect of thalassaemia and neonatal jaundice on the patterns. Colloids Surf. A.

[12] James SH, Kish PE, Sutton TP (2005). Principles of bloodstain pattern analysis: theory and practice.

[13] Smith FR, Brutin D (2018). Wetting and spreading of human blood: Recent advances and applications. Curr. Opin. Colloid Interface Sci.

[14] Attinger D, Moore C, Donaldson A, Jafari A, Stone HA (2013). Fluid dynamics topics in bloodstain pattern analysis: Comparative review and research opportunities. Forensic Sci. Int..

[15] Wendy JK, Barie G, Koen WJ, Bowers CM (2017). Chapter 9 - bloodstain pattern analysis. Forensic Science Reform.

[16] Laan N, Smith F, Nicloux C, Brutin D (2016). Morphology of drying blood pools. Forensic Sci. Int..

[17] Smith F, Nicloux C, Brutin D (2020). A new forensic tool to date human blood pools. Sci. Rep..

[18] Laan N (2019). The influence of coagulation on the drying dynamics of blood pools. Forensic Sci. Int..

[19] Ramsthaler F, Schlote J, Wagner C, Fiscina J, Kettner M (2016). The ring phenomenon of diluted blood droplets. Int. J. Legal Med..

[20] Laber T, Epstein B (2001). Substrate effects on the clotting time of human blood. Can. Soc. Forensic Sci. J..

[21] Schindelin J (2012). Fiji: an open-source platform for biological-image analysis. Nat. Methods.

[22] Thiriet M (2007). Biology and Mechanics of Blood Flows: Part II: Mechanics and Medical Aspects.

[23] Lowe, G. & Barbenel, J. Plasma and blood viscosity. *Clin. Blood Rheol.* **1**, (1988).

[24] Harkness J, Philips MJ (1981). Recording the plasma viscosity (a clinical pathology test) at a standard temperature. Bibl. Anat..

[25] Chao TC, Trybala A, Starov V, Das DB (2014). Influence of haematocrit level on the kinetics of blood spreading on thin porous medium during dried blood spot sampling. Colloids Surf. A.

[26] Rosina J (2007). Temperature dependence of blood surface tension. Physiol. Res..

[27] Quetzeri-Santiago MA, Castrejón-Pita AA, Castrejón-Pita JR (2019). The effect of surface roughness on the contact line and splashing dynamics of impacting droplets. Sci. Rep..

[28] Wang J (2015). Surface structure determines dynamic wetting. Sci. Rep..

[29] Cazabat A, Stuart MC (1986). Dynamics of wetting: effects of surface roughness. J. Phys. Chem..

[30] Windberger, U. *et al.* The effect of hematocrit, fibrinogen concentration and temperature on the kinetics of clot formation of whole blood. *Clin. Hemorheol. Microcircul.* **1–15**, (2020). 10.3233/CH-190799 32390608

[31] Wongcharee K, Brungs M, Chaplin R, Hong Y, Sizgek E (2004). Influence of surfactant and humidity on sol-gel macroporous organosilicate coatings. J. Sol-Gel. Sci. Technol..

[32] Pauchard L, Parisse F, Allain C (1999). Influence of salt content on crack patterns formed through colloidal suspension desiccation. Phys. Rev. E.

[33] Bohn S, Pauchard L, Couder Y (2005). Hierarchical crack pattern as formed by successive domain divisions. Phys. Rev. E.

