# Supplementary material for: Phase separation during blood spreading
Source: Sci Rep. 2021 Jun 3;11:11688. doi: 10.1038/s41598-021-90954-5 (PMC8175381; doi:10.1038/s41598-021-90954-5)
Supplement: Supplementary file 1 — Supplementary Information 1. [file 41598_2021_90954_MOESM1_ESM.docx]

**S1. Video.** Time lapse of a drying pool of blood (*mi* = 4.83 *g*, *hct* = 41.5 %) on varnished wooden floors at 21 °C with a relative humidity of 60 %, showing spreading and serum separation
